# Severity Classification of Parkinson’s Disease via Synthesis of Energy Skeleton Images from Videos Produced in Uncontrolled Environments

**DOI:** 10.3390/diagnostics14232685

**Published:** 2024-11-28

**Authors:** Nejib Ben Hadj-Alouane, Arav Dhoot, Monia Turki-Hadj Alouane, Vinod Pangracious

**Affiliations:** 1 Electrical and Computer Engineering Department, American University in Dubai, Dubai P.O. Box 28282, United Arab Emirates; vpangracious@aud.edu; 2 Columbia College, Columbia University, New York, NY 10027, USA; arav.dhoot@columbia.edu; 3 College of Computer Science, King Khalid University, Abha 62521, Saudi Arabia

**Keywords:** Parkinson’s diagnosis, classification, CNN, ViT, ResNets, Skeleton Energy Image (SEI)

## Abstract

**Background/Objectives:** Parkinson’s Disease is a prevalent neurodegenerative disorder affecting millions worldwide, primarily marked by motor and non-motor symptoms due to the degeneration of dopamine-producing neurons. Despite the absence of a cure, current treatments focus on symptom management, often relying on pharmacotherapy and surgical interventions. Early diagnosis remains a critical challenge, particularly in underserved areas, as existing diagnostic protocols lack standardization and accessibility. This paper proposes a novel framework for the diagnosis and severity classification of PD using video data captured in uncontrolled environments. **Methods:** Leveraging deep learning techniques, our approach synthesizes Skeleton Energy Images (SEIs) from gait sequences and employs three advanced models—a Convolutional Neural Network (CNN), a Residual Network (ResNet), and a Vision Transformer (ViT)—to analyze these images. Our methodology allows for the accurate detection of PD and differentiation of its severity without requiring specialized equipment or professional oversight. The dataset used consists of labeled videos capturing the early stages of the disease, facilitating the potential for timely intervention. **Results:** The four models performed very accurately during the training phase. In fact, an accuracy higher than 99% was achieved by the ViT and ResNet models. Moreover, a lesser accuracy of 90% was achieved by the CNN five-layer model. During the test phase, only the best-performing models from the training experiments were tested. The ResNet-18 model has achieved a 100% accuracy. However, the ViT and the CNN five-layer models have achieved, respectively, 99.96% and 96.40% test accuracy. **Conclusions:** The results demonstrate high accuracy, highlighting the framework’s capabilities, and in particular the effectiveness of the workflow used for generating the SEI images. Given the nature of the dataset used, the proposed framework stands to function as a cost-effective and accessible tool for early PD detection in various healthcare settings. This study contributes to the advancement of mobile health technologies, aiming to enhance early diagnosis and monitoring of Parkinson’s Disease.

## 1. Introduction

Parkinson’s Disease (PD) is an incurable neurodegenerative condition affecting an estimated 10 million individuals worldwide [1]. The onset of PD is characterized by a decline in muscular function, primarily due to the degeneration of dopamine-producing neurons in the brain. While the exact cause remains uncertain, a combination of genetic, environmental, and lifestyle factors is believed to contribute to its onset. Aging is a significant risk factor, with most cases occurring in individuals over the age of 60.

The primary symptoms of PD include tremors, bradykinesia (slowness of movement), rigidity, and postural instability. Beyond these motor symptoms, PD can also lead to non-motor symptoms such as cognitive impairment, depression, and sleep disturbances, complicating the overall clinical picture [2].

Current treatments for PD focus on managing symptoms, as there is no cure for the condition. Common strategies include pharmacotherapy, which often involves medications designed to replenish or mimic dopamine levels in the brain to alleviate motor symptoms. Additionally, deep brain stimulation has emerged as a surgical intervention for individuals with advanced PD. However, physical therapy is considered the gold standard for non-invasive management, particularly when initiated early in the disease course. Engaging in physical therapy can help reduce tremor severity and improve the overall quality of life for patients.

Diagnosing PD presents significant challenges, as there is currently no universal protocol. Physicians rely on a combination of laboratory tests, blood analyses, and physiological assessments to render a diagnosis. Unfortunately, many patients do not receive a PD diagnosis until the disease has progressed to advanced stages. This diagnostic challenge is particularly acute for residents in rural and remote areas, who often have limited access to specialized healthcare resources. Furthermore, non-Parkinsonian gaits may sometimes be misclassified as Parkinsonian, leading to inappropriate therapies and medications.

With the recent advancement in computational power and increased access to technology, research is being conducted to develop computer-based solutions assisting in the diagnosis of PD [3]. These solutions aim to develop tools accessible in diverse environments, to facilitate its early and accurate detection. For example, mobile systems hold promise for assisting healthcare professionals with the diagnosis and management of PD. These technologies could offer tools for preliminary assessments, aiding in the early identification of symptoms and facilitating more timely interventions.

Advanced algorithms and artificial intelligence can analyze vast and diverse datasets, aiding in the identification of hidden patterns associated with PD. In fact, research for the effective application of machine learning (ML) and deep learning (DL) to develop systems to help with the diagnosis of PD is ongoing. Over the past decade, multiple ML and DL systems have been developed to detect PD based on multiple media datasets [3].

In particular, early systems have been developed for the diagnosis of PD based on the analysis of handwriting [4], speech [5], and other types of data. More modern systems have leveraged video data and focused on using feature engineering techniques combined with ML algorithms [6]. Most of these, however, require video datasets developed in controlled environments or under the supervision of professionals. Obviously, this limits their availability and reduces their impact for the above stated cause linked to early diagnosis and availability in environments with limited resources.

The methodology developed in this paper for the diagnosis and severity detection of PD is based on video data produced mostly in uncontrolled environments. These videos involve the subjects simply walking in different situations and turning in different directions. In fact the dataset we use in this paper is the same used in [7] and has been collected from various open sources. The resulting gaits are automatically analyzed to generate a PD diagnosis. This diagnosis technique, based on a person’s gait, is known to be reliable and is commonly used by neurologists and physicians [8].

Currently, most video-based systems necessitate a significant level of human intervention for both recording the videos and generating the diagnoses [9]. For instance, the systems described in [10,11] require the placement of physical sensors on the subjects, while [12] relies on digital markers applied to the subject’s body. Consequently, these systems are not more accessible than traditional methods that do not utilize video data. Furthermore, even studies employing video data combined with various feature engineering techniques and ML, without needing specialized artifacts, mandate that videos be captured in controlled environments [13]. This requirement enhances the effectiveness of their feature engineering algorithms [14].

In contrast, the framework developed in this paper employs modern deep learning techniques to facilitate accurate detection and severity classification of Parkinson’s Disease. For configuring our deep learning models, we utilize a generic video dataset recorded in uncontrolled environments, without any specific directions or professional assistance [7]. This dataset is labeled with four severity levels according to the classification standard set by the Unified Parkinson’s Disease Rating Scale (UPDRS) [15]. It includes videos of subjects in the early “mild” and “moderate” stages of the disease, allowing our framework to effectively capture and distinguish these early stages of PD. Thus, the methodology developed in this paper is designed to function in various environments using standard cameras, without requiring professional intervention. This enables doctors to use it as a diagnostic aid in their offices or while remotely connecting with patients in their homes, even in remote areas. Furthermore, the proposed framework, subject to the availability of an adequate database, can be applied to detect other types of gait abnormalities related to neurological diseases. Many neurological diseases, such as Alzheimer’s disease, cerebellar ataxia, Huntington’s disease, etc., impact the human gait, yielding various gait disorders [16].

More specifically, the framework includes a workflow for pre-processing the video dataset that uses DL-based techniques to synthesize Skeleton Energy Images (SEIs) [17]. These SEIs involve sequences of images capturing the gaits of the respective subjects. Three deep learning models—a Convolutional Neural Network (CNN), a Residual Network (ResNet), and a Vision Transformer (ViT)—are trained and applied to the classification task. All models demonstrate effective performance with the workflow, achieving high accuracy in diagnosing PD and classifying its severity.

The primary objective of this study is to leverage deep learning techniques in a computationally efficient manner to develop a system capable of detecting Parkinsonism from simple video data. Considering the main use case of wide availability and early detection, our system is cost-effective and less complex compared to existing methods. By optimizing computational efficiency, this research aims to contribute to the advancement of mobile health technologies, providing a cost-effective and widely accessible solution for the early detection and monitoring of Parkinson’s Disease.

This paper is organized as follows: The next section presents an extensive literature review highlighting significant works in the field. Section 3 introduces and describes the dataset used in our study. Section 4 details the workflows of our methodology, particularly discussing the pre-processing applied to the dataset and the synthesis of SEIs using human pose estimation techniques. Section 5 evaluates and discusses the effectiveness of the deep learning models employed for the classification task. Section 6 presents our comprehensive experimental study, detailing our results and evaluations with thorough discussions. Finally, Section 7 concludes this paper.

## 2. Literature Review

Numerous approaches have been explored in the literature for the automated detection and diagnosis of Parkinson’s Disease. Many studies have employed various features, including handwriting [4], eye movements [18], and biomarker data [19]. However, methods utilizing image and video analysis are particularly effective, as they provide a direct visual representation of the motor symptoms associated with PD, closely resembling a doctor’s diagnosis.

A review in [20] highlighted the connection between human gait and PD. Early-stage PD is characterized by movement irregularities, reduced arm swing, and limited motion in the lower limb joints (ankle, knee, and hip). As the disease progresses to mild and moderate stages, patients exhibit more pronounced impairments, such as stooped posture during walking and fragmented turning. In advanced stages, symptoms like freezing gait and severe postural issues become evident. The severity of PD is assessed using UPDRS, which rates gait on a scale from 0 to 4, where 0 indicates a normal gait and 4 signifies a complete lack of movement. Consequently, vision-based gait analysis techniques are essential for the automated diagnosis of Parkinson’s Disease. Recent advancements in computing hardware and the development of more efficient deep learning and machine learning (ML) models have accelerated investigations into automated PD diagnosis using video data [21].

Many studies combine feature extraction from videos and images with ML techniques to enhance PD detection. In [14], a computer vision technique is proposed to differentiate between normal and Parkinsonian gaits. They employed an a Region-Based Convolutional Neural Network (R-CNN) to extract human silhouettes from RGB video frames, generating Gait Energy Images (GEIs) for further processing. This method, evaluated on a dataset of 228 normal gait videos and 73 PD gait videos collected in a controlled environment, achieved an accuracy of 97.33%. Similarly, in [13], a 2D pose estimation is utilized to extract gait features and identify four PD stages using various ML algorithms, including Support Vector Machine (SVM), achieving an accuracy ranging from 96% to 99%.

A similar approach is proposed in [21], which involves extracting 3D key points for significant body joints using OpenPose software. This method allows for the extraction of features characterizing the gait of individuals with Parkinson’s Disease, healthy older adults, and individuals with multiple sclerosis (MS). The study employed Residual Neural Networks (RNNs) and 1D Convolutional Neural Networks (CNNs), achieving an accuracy of 78.1%. The dataset comprised locally collected videos from 33 participants, including 10 with MS, 9 with PD, and 14 healthy individuals.

Four additional leading studies in the field of PD diagnosis utilize video data and feature extraction approaches. The first, presented in [7], analyzes gait patterns from video recordings using an ML model, namely, Random Forest (RF), on key-point features obtained via AlphaPose, a human pose estimation model. This study highlights the advantages of human pose estimation for computationally efficient feature extraction. Notably, it employs a self-collected dataset consisting of videos sourced from uncontrolled environments in open-access domains, which is also used in this study.

The second approach, as proposed in [22], focuses on gait features to identify Parkinson’s stages in individuals with dementia, assessed using the UPDRS gait scale. This method begins with pose estimation, followed by extracting body joint coordinates using MediaPipe for gait analysis. Various machine learning algorithms, including Extra Trees, achieved an accuracy of 90.1%. The dataset included videos from 14 elderly individuals exhibiting diverse gait patterns.

The third method leverages motion features from 3D skeleton estimation, particularly using pelvic position coordinates to detect freezing gait in patients. The fourth study [23] focuses on detecting Freezing of Gait (FoG), a specific PD symptom, in uncontrolled clinical environments. This study combines a rule-based model utilizing pelvic coordinates to detect walking stops with a machine learning model based on a 1D-CNN linked to a Long Short-Term Memory (LSTM) network. This hybrid approach enables accurate FoG detection from videos recorded during routine clinical practice, even with multiple individuals present. The system demonstrated high reliability, with an intraclass correlation coefficient ranging from 0.75 to 0.94 compared to expert annotations. This innovation offers potential for monitoring FoG in home environments using standard video cameras, such as those on smartphones. However, challenges remain in cluttered scenes and variations in lighting or camera angles that may impact detection accuracy.

With advancements in computing capabilities, including powerful GPUs, video serves as a promising medium for PD detection [24]. However, the literature on the intersection of deep learning approaches and video data for PD detection and severity classification remains sparse [25]. A recent comprehensive study in this area [24] highlighted the necessity and importance of such tools, discussing challenges like the need for quality video datasets. It noted that deep learning solutions for PD diagnosis based on video data often exhibit significantly lower accuracy compared to traditional machine learning approaches. Furthermore, many proposed video-based systems require access to specialized software and hardware, posing accessibility barriers for widespread adoption in normal clinical settings or at home.

In [26], a complex, entirely DL-based approach is proposed for the automated diagnosis of gait impairments in PD. Silhouettes and 2D skeletons are extracted from gait videos, from which pose graphs are constructed. Long-Term Gait Energy Images (LT-GEIs) are computed from silhouettes, defined as a composite image of GEIs extracted from three gait cycles. The two streams of pose graphs and LT-GEIs are fused using a Spatio-Temporal Graph Convolutional Network (STGCN), achieving an overall classification accuracy of 71.25% on a controlled dataset comprising 54 PD patients and 26 healthy subjects.

Additionally, [27] introduced a novel dataset called GAIT-IT, consisting of videos captured in controlled settings, featuring 21 patients displaying five different gait types, not limited to PD, with only two severity levels. The authors noted the lack of existing video data and surveyed four existing small datasets, primarily composed of synthesized video data. They discussed two key methods for generating synthetic images from videos. The first, originating in [28], involves synthesizing silhouette images from video frames and aggregating gait sequences into Gait Energy Images (GEIs). The second, more recent approach discussed in [17], utilizes OpenPose to synthesize skeleton frames from images, aggregating gait sequences into Skeleton Energy Images (SEIs). Furthermore, this study compared the SEI approach with the GEI approach using a CNN on one of the small available datasets, concluding that SEIs yield better results.

Lastly, the Gait Abnormality Video Dataset (GAVD), a relatively large dataset containing 1,874 normal, abnormal, and pathological gait sequences, not all related to PD, is discussed in [29]. The GAVD includes clinically annotated RGB data sourced from publicly available online content, comprising more than 400 subjects captured in various settings, such as urban uncontrolled outdoor environments and hospital clinics. This dataset has been employed with Temporal Segment Networks and SlowFast networks, achieving over 90% accuracy in abnormality detection.

In summary, our literature review highlights the scarcity of raw video datasets for the automated diagnosis of Parkinson’s Disease. The few available datasets have been developed in controlled settings and do not necessarily specialize just in Parkinson’s, and often encompass multiple neurological diseases. Moreover, the publicly available datasets primarily consist of synthetic artifacts (silhouettes, skeletons or key points) rather than actual video recordings. Most datasets used in the studied papers are collected locally. Thus, it is practically difficult and even impossible to compare the state-of-the-art findings as they are not related to our dataset or Parkinson’s Disease.

While deep learning approaches are gaining traction for the diagnosis of Parkinson’s Disease, due to the above hurdles, machine learning methods based on feature engineering techniques remain the most prevalent. Indeed, few papers in the current state of the art have proposed approaches based on deep learning models. However, gait analysis combined with deep learning techniques can be interesting in understanding and interpreting gait abnormalities, allowing an efficient prediction and severity classification of Parkinson’s Disease and also the distinguishing between neurological diseases. It is very important to notice that the choice of the best deep neural network architecture may depend on the size of the dataset and the aims of the research. The dataset size and the video recording context are important factors in selecting the appropriate deep neural network architecture.

## 3. Dataset Description

The dataset utilized for this study is the same as the one used in [7]. It comprises 292 videos divided into four classes: **normal, mild, moderate, and severe**. In fact, these classes correspond to stages 0 to 3 of the UPDRS [15] standard five stages of PD. It is generally viewed that in Stage 4 the patient is unable to move, and this is obviously why it is not represented in the dataset.

The videos in our dataset were obtained from open-access domains and included recordings of 167 subjects, comprising 93 healthy individuals and 74 with Parkinson’s Disease. Notably, the videos of patients with PD were primarily captured during therapeutic sessions. To ensure privacy, an anonymized version of the dataset was released to us. This anonymization was performed in two ways.

**Figure 1:**
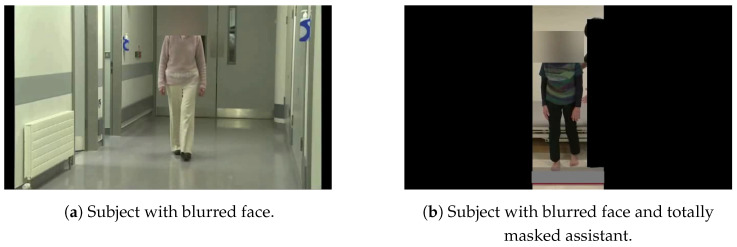
Different blurring situations.

The first subset of videos featured only the subjects, who were visible in all frames, as illustrated in the left image of Figure 1, where the corresponding subject’s face is blurred. The second subset involved severe cases of PD, where medical assistance was required. In this subset, any medical personnel present, in addition to the subject, were fully masked, as shown in the right image of Figure 1.

The dataset encompasses recordings captured both indoors and outdoors, under various environmental and lighting conditions. Additionally, different camera angles relative to the subject’s position were utilized. This diversity presents both a challenge and a robust foundation for training the image classification algorithms we employed, as it offers sufficient variability in the visual inputs. Considering these factors, it is clear that the dataset effectively supports the development and evaluation of algorithms and applications aimed at the rapid, non-invasive diagnosis and severity assessment of Parkinson’s Disease.

One can easily envision a scenario where a camera is placed in the office of a medical practitioner, such as a neurologist, equipped with a tool that provides insights into the patient’s condition as they walk in, even before the session begins.

**Figure 2:**
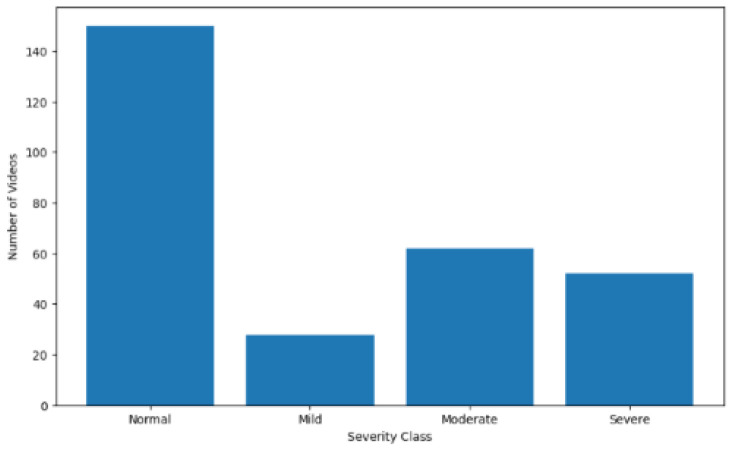
Distribution of videos by severity of Parkinson’s Disease (by class label).

Examining the distribution of the four classes in the dataset, as shown in Figure 2, reveals a notable imbalance. The “normal” class, representing healthy subjects, contains significantly more videos than the other classes. Moreover, within the categories of subjects labeled as having varying degrees of PD, the “mild” class has only half as many videos as each of the other two classes. At this stage, the data remain in their raw form; as we will discuss later, several techniques will be employed to introduce balance among the classes.

## 4. Methodology and Workflow

Our methodology involves generating Skeleton Energy Images (SEIs) from skeleton images (SIs). These SIs are obtained through Human Pose Estimation (HPE) techniques, applied to our dataset following appropriate pre-processing. The goal is to create a training and testing dataset composed of SEIs [26], which is subsequently used to parameterize the various models proposed in this paper for the identification and diagnosis of Parkinson’s Disease.

As a matter of fact, we generate two SEI datasets. The first is designated as the normal SEI dataset, and the second is designated as the key-frames SEI dataset. For simplicity purposes, we chose, in what follows, to describe in detail the process for generating the first, normal-SEI dataset. The last subsection details the modifications in processing, which are minor, to also obtain the second key-frames SEI dataset.

This section details the two workflows needed for producing the normal SEI dataset from the original dataset, consisting of the repository of videos described in the previous section. The first subsection describes the pre-possessing workflow, yielding subsets of sequential frames, form each video. The next subsection deals with using HPE, yielding the skeleton frames. The last subsection describes how several skeleton images are combined into an SEI frame, thereby producing the target SEI dataset.

### 4.1. Dataset Pre-Processing

The main objective of the pre-processing is to enable the computation of the Skeleton Energy Images. Moreover, the videos in the original dataset were recorded in uncontrolled environments but do not carry any noise, so a denoising process is not needed for this dataset.

To generate a Skeleton Energy Image (SEI), the required skeleton images (SIs) must be derived from successive frames within a single video. Additionally, these frames should either capture the subject facing a single direction or transitioning between directions. This requirement helps in minimizing any noise during the training phase, which could negatively impact the generalization capabilities of our target models. In order to efficiently produce such SIs, the videos in our original dataset described in the previous section need to be processed through two workflows.

**Figure 3:**
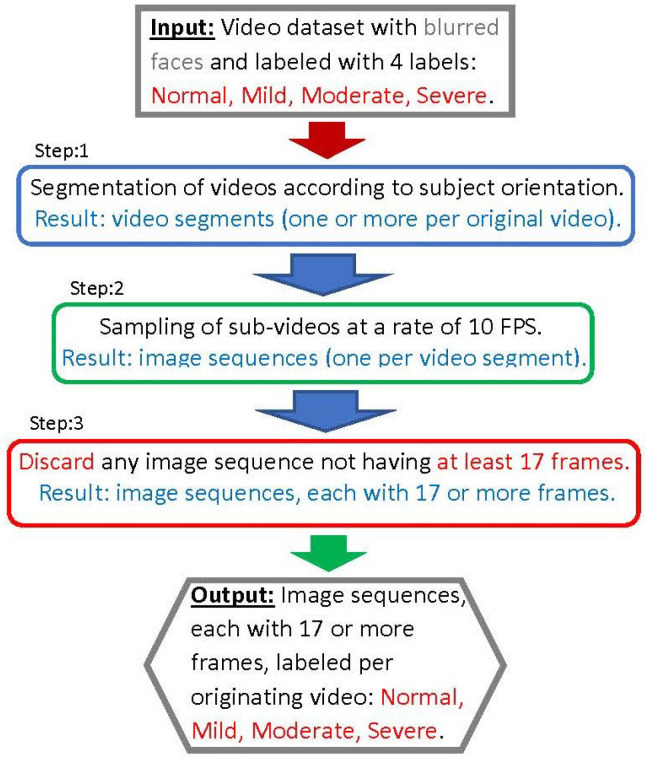
Pre-processing workflow producing sequences of images from videos.

The steps of the first workflow, illustrated in the diagram shown in Figure 3 and designated as the pre-processing workflow, are described in this section. Note that the steps framed in blue are performed manually; those framed in green are automated (through a script); and the steps framed in red involve discarding certain information, such as sets of frames.

The first step involves manually segmenting each video into one or more clips. Each clip captures the subject in one of four possible orientations: (1) facing forwards, (2) facing backwards, (3) facing sideways, or (4) transitioning between orientations. In the second step, each video segment is sampled at a rate of 10 frames per second, resulting in correspondingly labeled frame/image subsets from the segments.

The third step involves discarding segments that yield fewer than 17 frames after sampling. Frames from each segment are kept separately and labeled according to their originating sequence and/or video. It is important to note that most videos result in frame sequences that capture the subject in the various orientations listed above. Proper segmentation of the videos is crucial for generating a relevant and de-noised SEI dataset [30]. This dataset is used in the subsequent sections of this study.

**Figure 4:**
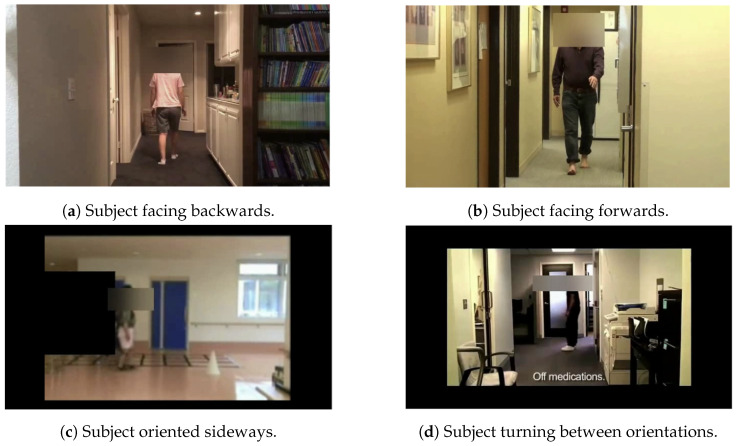
Representative frames from four possible video segmentation situations.

Figure 4 displays representative frames depicting subjects in the four described orientations.

### 4.2. Human Pose Estimation

There is a well-established consensus in the literature on ML-based diagnosis that understanding and analyzing the movements and trajectories of key human joints (such as the arms, legs, and shoulders) is crucial. Our proposed method for the automatic diagnosis of Parkinson’s Disease relies on extracting these key points, which represent the joints, from frames obtained after Step 3 of our pre-processing workflow. This extraction is performed automatically using Human Pose Estimation.

**Figure 5:**
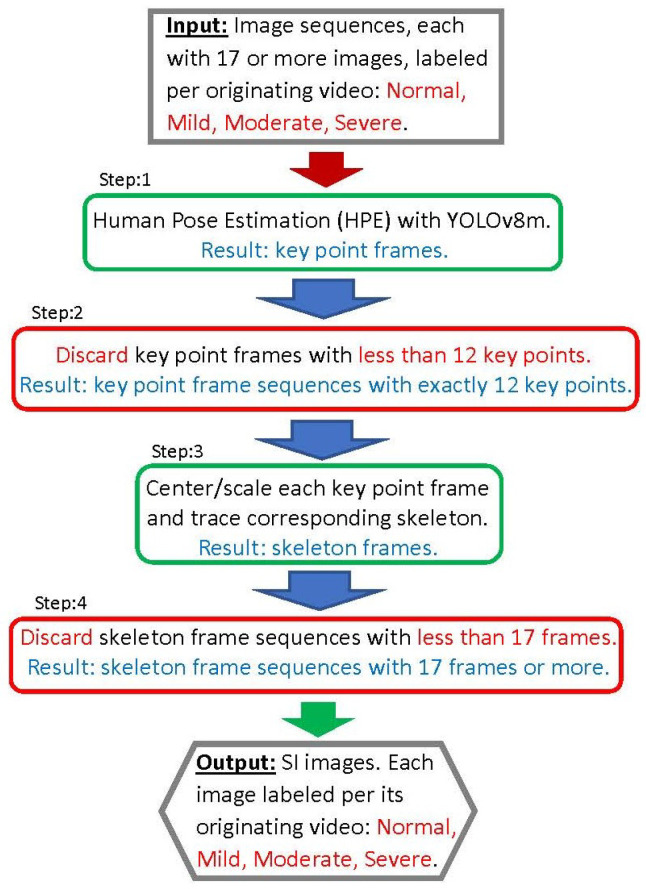
SI workflow producing sequences of skeleton images.

This subsection details our second workflow, illustrated in Figure 5, and designated as the SI workflow. Its objective is to apply humain pose estimation on the sequences of images resulting from the pre-processing workflow of the previous subsection. This results in sequences of SIs corresponding the the image sequences. Note that the steps of the workflow have frame colors indicating their types of function as indicated in the previous subsection.

The main tool used in this SI workflow is the pre-trained and highly effective YOLOv8m HPE model, which comprises 26.4 million parameters [31]. In step 1 of our SI workflow, each input image is processed by the YOLOv8m model to produce a frame with the corresponding key points. If this operation is successful, the following 12 key points are extracted [31]: right shoulder, left shoulder, right elbow, left elbow, right wrist, left wrist, right hip, left hip, right knee, left knee, right foot, and left foot.

**Figure 6:**
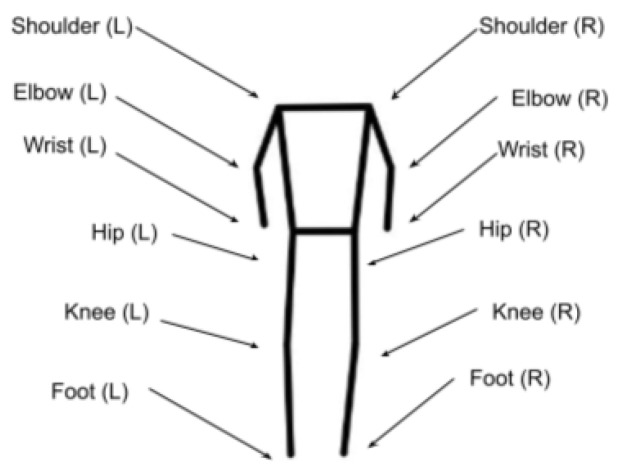
List of joints/key points returned by HPE.

Figure 6 illustrates the joints corresponding to a human skeleton, returned as key points by the HPE model. To understand how the HPE model works, we designate these key points with the following notation,

$$\{\left(x_{1},y_{1},p_{1}\right),\left(x_{2},y_{2},p_{2}\right),\ldots ,\left(x_{12},y_{12},p_{12}\right)\},$$

where $x_{i}$ and $y_{i}$ denote the 2D coordinates of the *i* the key point, and $p_{i}$ represents the confidence score of the corresponding key point’s 2D coordinates. A key point coordinate is considered valid and is retained if it corresponding probability score is $p_{i}>0.5$. In fact, in step 2 of our SI workflow, each image that results in fewer than 12 key points is discarded.

We should also recall that each key point frame produced in step 2 corresponds to an image that is part of a sequence of images, and this image information regarding its order within the sequence and its class label, corresponding to its originating video. Obviously, the resulting key point frames keep this information and forward it to corresponding results in the SI workflow.

Step 3 in our SI workflow, consists of two sub-steps. The first sub-step involves centering and scaling the 12 key points in each frame to a height of 360 pixels. This process is crucial for maintaining spatial consistency and preventing distortions in the resulting dataset that could arise from variations in the relative positions of subjects within the original videos.

The second sub-step involves tracing a skeleton from the 12 key points, thereby converting each centered and scaled key point frame into an SI, i.e., skeleton image. This skeleton image/frame represents a headless human skeleton of the corresponding subject, as heads and faces were blurred in the original video dataset to protect the anonymity of the subjects.

**Figure 7:**
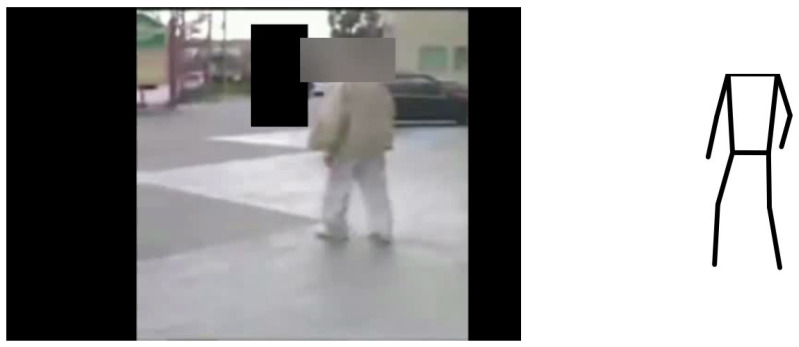
Image from video with corresponding SI.

Figure 7 illustrates a sample image originating from one of the videos alongside its corresponding SI.

In step 4 of the SI workflow, we discard any sequence of SIs not having at least 17 SIs. Obviously, some sequences resulting from a corresponding input sequence of 17 or more images are discarded, but some of these images resulted in key point frames that were discarded in step 2 as they had less than the 12 required key points.

### 4.3. Energy Images

The concept of the Skeleton Energy Image (SEI), as utilized in this study, is based on the energy image concept outlined by [27]. An energy image is essentially the average of multiple consecutive frames, designed to extract key features from the movements and joint trajectories of the subject. Additionally, it accentuates and isolates these movements and trajectories, which facilitates the effective detection of early forms of PD [32].

While [27] used a variable number of frames to produce a single energy image, our study generates a Skeleton Energy Image (SEI) from each of the consecutive 17 SIs, which empirically corresponds to approximately 1.5 gait cycles. This approach aligns with analysis criteria developed in prior works that base effective PD detection on gait [20]. The technique employed is described by the following equation, where $E_{\left(i,j\right)}$ represents the pixel value at position (i,j) in the SEI. It is computed by summing the corresponding pixels of each of the 17 skeleton images and dividing this sum by 17:

$$E_{\left(i,j\right)}=\frac{1}{17}\sum_{n=1}^{17}S_{\left(i,j\right)}^{n}$$

**Figure 8:**
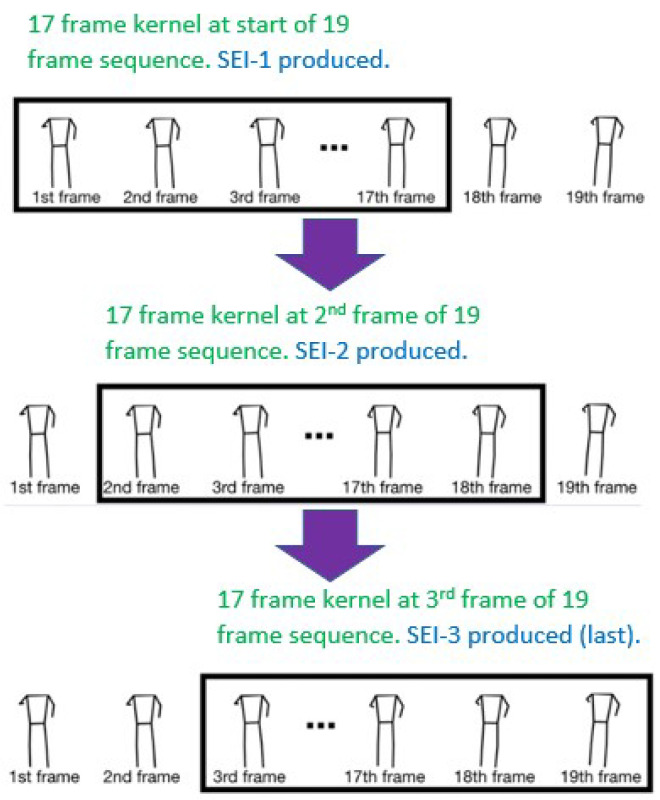
Rolling kernel used for creating SEI.

Our approach further differs from [27] by employing a rolling kernel to generate multiple SEIs from a sequence of skeleton images. This process is illustrated in Figure 8. When the sequence contains more than 17 skeleton images, the first 17 images are used as the kernel to produce the initial SEI. The kernel is then moved to start with the next image, and so on, until the last kernel captures the final 17 skeleton images in the sequence. In the case the sequence contains exactly 17 frames, obviously, only one SEI is produced from it. Moreover, recall that sequences with fewer than 17 frames were entirely discarded during the pre-processing stage.

In addition to augmenting the SEI dataset, this method increases the amount of information made available to our image classification models and enhances their generalization capability. This is because the same joint trajectory is captured by multiple SEIs, each starting from different coordinates.

### 4.4. Processing Differences for the Generation of the Key-SEI Dataset

It was noted earlier in Section 3 that, in fact, for the purposes of our experiments, described later, two SEI datasets were synthesized from our original dataset. The difference in the processing is not significant, and, for purposes of simplification, we deal with it in this subsection.

In fact, step 3 of the first pre-processing workflow (the sampling step) is executed in two different ways, leading to obtaining each of the two SEI datasets. This is described below.

A frame is classified as a key frame if it has a difference in value that is 1.5 times higher than the average of the previous 10 frames [33]. This method for selecting candidate frames to be examined was utilized within the context of emotion detection in [34]. The main objective was to avoid training the model on similar images.

The first frame in a video segment is always designated as a key frame; then, all other key frames following it are identified. For any key frame, the 16 frames following it are taken as part of its sequence. If fewer than 16 frames follow a key frame in the corresponding video segment, then no frame sequence starting with this key frame is formed. Therefore, a key-frame sequence has exactly 17 images, whereas the normal frame sequence, corresponding to the video segment, consists of all the frames. Moreover, a video segment may generate zero or more frame sequences.

For the rest of the steps, while key-frame sequences are treated separately from the normal frame sequences, the steps of the workflows are applied to both in the same way. Note, for example, that an SI key-frame sequence may result from one key-frame sequence, and in that case, one key-frame SEI is generated from it. However, if during the HPE step (step 2) of the SI-workflow, a key-point sequence is rejected because less that 12 key points were generated from the corresponding frame, then the entire key-frame sequence generates no SEI.

## 5. Selection of Deep Learning Models

In this section, we deal with the selection of a candidate set of deep learning models for the purpose of using them in the task of diagnosis of PD. This is in fact a task which involves the classification of the various videos in our original dataset, based on the synthesized SEI dataset produced from them. We have discussed in the previous two sections how the original dataset is processed and how these synthetic SEI images are produced from each corresponding video.

**Figure 9:**
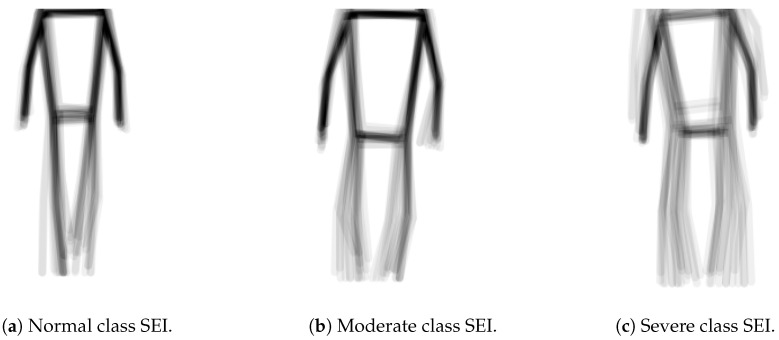
SEIs from front-facing subjects.

In our analysis of the synthesized SEI dataset, we concluded that effective classification requires a focus on the global aspects of SEI images rather than on specific detailed features. This is illustrated by the SEI images in Figure 9, which represent three classes from the dataset: normal, moderate, and severe. Notably, posture is a key differentiator; the normal class displays a more upright stance compared to the others. Additionally, the severe class is characterized by slow-moving feet. Overall, the normal SEI exhibits correct posture with sharp, fast limb movements, while the moderate and severe classes display slower movements and more stooped postures.

These observations prompted us to explore multiple models for the classification task rather than relying on a single architecture. In this study, we utilize and compare results from three prominent deep learning architectures: Convolutional Neural Networks (CNNs), Residual Networks (ResNets), and Vision Transformers (ViTs). We anticipate that the last two architectures will be particularly well suited to our classification objectives.

This section is organized as follows. The first three subsections reviewing the above-mentioned three deep learning architectures and give indications about the suitability of each architecture for our classification task. In the last section we summarize our evaluation and shed light on particular parameters that we shall be using for each model/architecture.

### 5.1. Convolutional Neural Network (CNN) Architectures

CNNs are a class of deep learning algorithm that operates on the principle of hierarchical feature learning [35]. Initially, the convolutional layers capture low-level features like edges and textures from the raw pixel data. As the data progress through the deeper layers, increasingly complex features and patterns are recognized, culminating in high-level representations of the input data.

CNNs are popularly used, in their 2D format, for tasks involving the classification of images. And since our SEIs can be considered as images showing a skeleton in a particular trajectory, it is only natural that we consider using CNNs for our classification task.

When designing the CNNs for this study, however, we pondered the fact that the SEIs, in fact, have relatively simple features, and, therefore, would go better with shallow CNNs. Moreover, along with the important convolutional, pooling, and fully connected layers that we included, we found it important to incorporate batch normalization and dropout layers following each of the above layers of the network. This allows the model to proceed directly and speedily to the relevant details due to its relatively shallow depth.

Batch normalization is used both in feed-forward and convolutionnal neural networks with two main advantages [36]. First, it helps to mitigate the problem of the internal co-variate shift of the network, where the distribution of the inputs to each layer can change during training, and thus has a stabilizing effect on the learning process. Second, it is believed to have a regularization effect, since it is computed over the batches and not over the entire dataset

Dropout is a also a regularization technique designed to prevent overfitting [37]. During training, dropout randomly sets a fraction of the activations to zero in a given layer, effectively preventing the network from relying too heavily on any particular neurons. This enables the network to develop redundant representations, thereby improving its ability to generalize to new, unseen data.

### 5.2. Residual Networks (ResNets)

ResNets, introduced in [38], address the degradation problem prevalent in deep neural networks, where increasing depth can lead to a higher training error. The key innovation of ResNets is the incorporation of residual learning through shortcut connections. These connections enable the network to learn residual functions relative to the inputs of each layer, rather than attempting to learn unreferenced functions directly.

Residual functions are defined as the difference between the desired output and the input to the layer, allowing the model to focus on learning the changes needed to reach the target output. Mathematically, if F(x) represents the desired mapping to be learned, the residual learning framework transforms this into learning the residual, i.e., F(x)−x. This allows the network to adjust its outputs based on how far the learned function deviates from the identity mapping and simplifies the optimization process.

This departure from CNNs also facilitates the flow of gradients during backpropagation, alleviating the vanishing gradient problem and allowing for the effective training of networks with hundreds or even thousands of layers. The residual connections provide a direct path for gradients to flow back through the network, enabling deeper architectures to maintain performance without suffering from the typical pitfalls associated with increased depth.

The architecture of a ResNet is characterized by its use of residual blocks, which combine convolutional layers with batch normalization and ReLU activation functions. Within these blocks, the shortcut connections bypass one or more layers, allowing the original input to be added to the output of internal convolutional layers. This additive operation empowers the network to learn identity mappings, ensuring that performance does not degrade as depth increases.

By explicitly allowing layers to copy their inputs directly to their outputs, ResNets streamline the optimization of very deep networks. This structure encourages the layers to focus on learning adjustments to the identity function rather than recreating outputs from scratch. Consequently, even if some layers learn suboptimal transformations, the overall network can still preserve performance, as the identity mapping is easily accessible through the shortcut connections.

In summary, the concept of residual functions is central to the success of ResNets, enabling deeper networks to train effectively by focusing on learning modifications to established patterns rather than starting from zero. This not only enhances the efficiency of training but also improves the generalization capabilities of deep learning models in various applications.

### 5.3. Vision Transformers (ViTs)

Vision Transformer models (ViTs), introduced in [39], represent a significant paradigm shift in image recognition tasks by leveraging the transformer architecture originally designed for Natural Language Processing (NLP) [40]. In this innovative approach, ViTs partition an image into fixed-size patches, treating these patches similarly to sequences of tokens in NLP, akin to words in a sentence. This methodology utilizes the self-attention mechanism inherent in transformers, which effectively models complex, long-range dependencies and relationships between different parts of the image. This alleviates the loss of detail that can occur early in Convolutional Neural Networks (CNNs), where the focus is predominantly on local features and their synthesis.

The ViT architecture functions by linearly embedding the fixed-size patches into vector representations, augmented by positional embeddings that retain spatial information. This allows the model to understand the arrangement of patches within the original image. These embeddings are then processed through multiple layers of Transformer encoders, each comprising multi-head self-attention mechanisms and feed-forward neural networks. Unlike CNN layers, which prioritize local interactions, the self-attention mechanism in ViTs enables the model to dynamically attend to various parts of the image, capturing global context effectively.

Recent studies have shown that ViTs outperform traditional CNNs [39,41] across various benchmarks, particularly when ample data and computational resources are available. The capacity of ViTs to leverage large datasets enhances their learning, making them particularly powerful for complex image recognition tasks. As a result, they are increasingly adopted in state-of-the-art image classification applications, demonstrating their potential to redefine the landscape of computer vision.

### 5.4. Evaluation of the Architectures

To understand the performance variations related to the number of learnable parameters for each type of model/architecture and their adaptability our task, we conducted preliminary experiments. These simple experiments allowed us to determine the appropriate depths to be used with each model type.

For the CNN architecture, we selected the 5-layer and 6-layer configurations; for the ResNets, we selected 18-layer and 34-layer variants; and, for the ViTs, we selected three sizes, the base size, the small size, and the tiny size.

Notably, the selected model depths/sizes are shallower than those typically used in conventional studies. For example, while ResNet-50 is widely used for image classification tasks, we only employed ResNet-18 and ResNet-34. Similarly, although ViT-Base is commonly used as the baseline model, we used it as the largest ViT model in our study. The SEI images that we attempt to classify, as shown in Figure 9, do not have particularly complex or numerous features. Thus, the use of shallower models enhances computational efficiency and makes the models more suitable for deployment on smartphones and other devices without dedicated GPUs. This consideration makes the models trained in this study suited for this intended use case, particularly in developing nations, thereby ensuring broader accessibility and usability. Moreover, our expectations were realized in this preliminary study as the ResNets and ViT models we used outperformed the CNNs. It also became clear that that increasing the depth of the CNN model does not improve its results. This shall be addressed in more details in the next section.

## 6. Experimental Design, Results, and Discussion

In this section, we discuss the experiments that we utilize to test the models extensively. Specifically, we describe our rationale in designing the experiments and the specific parameters that bound our experiments, and, finally, this section ends by discussing the results of each of the experiments in great detail. We have an in-depth conversation about what the results entail and offer insights about which model might perform depending on the application.

### 6.1. Design of the Experiments

Based on our preliminary study, discussed in the previous section, we have selected the architectures and corresponding models used in the main comprehensive study that we conducted and discuss them in this section. The models involved are named as shown in Table 1.

In fact, we systematically conducted four experiments and evaluated the accuracy of each trained version of the selected models for the task of PD diagnosis and classification. These experiments used the two SEI datasets composed of Skeleton Energy Images and synthesized through the process described in the previous sections from our original dataset composed of videos. We recall that both SEI datasets, as is the case for the original dataset (see Figure 9), are imbalanced with respect to the four classes. So, a third, class-balanced dataset is produced from the normal SEI dataset using the random oversampling technique [42]. With oversampling, randomly selected images in the underrepresented classes were artificially duplicated within the respective class subset to achieve class balancing. Therefore, a total of three datasets, given in Table 2, are used for our comprehensive study.

We should note that while the original dataset is composed of 292 videos, they were used, with the workflow presented in Section 4.3, to synthesize 17,038 SEI images. Each SEI is computed using a rolling kernel with size 17 frames over one video subsequence (see Figure 8). The sizes of the normal SEI and balanced normal SEI datasets shown in Table 2 are relatively large to enable good learning of the models and to provide them with high generalization ability. However, the sizes of the key-frame SEI dataset is relatively small, which may limit the models’ generalization ability. Moreover, as we have mentioned in the literature survey section, raw video datasets in this domain are scarce and not available publicly [27,29]. In fact, the dataset we use in this paper [7] is among the largest.

All SEI datasets are split into respective training, validation, and testing sub-datasets using an 8:1:1 ratio via random sampling. The contents of the respective sub-datasets remained consistent throughout the four experiments. Moreover, we recall that the label associated with each SEI image in the training, test and validation SEI sub-datasets falls into one of four classes: normal, mild, moderate, and severe. In fact, this is the same label associated with the corresponding video from the original dataset from which the SEI image is synthesized/generated.

The four experiments were divided into two distinct categories. The first category comprised two experiments aimed at fine-tuning the training techniques to optimize model performance. The second category involved testing the trained models under diverse conditions to assess their robustness and generalization capabilities. The last category of experiments, is crucial for understanding how well the models performed under adverse scenarios; this underlined their ability in maintaining their corresponding accuracies across all situations.

The following describes the four experiments which we conducted for the purposes of our comprehensive study.

- **Experiment 1:** The training sub-set corresponding to the normal SEI dataset is used for the training all the models given in Table 1. The class distribution of the SEI images were proportional to those given in Figure 9. The model was evaluated on the corresponding test subset (for the normal SEI dataset).
- **Experiment 2:** The training on all the models given in Figure 9 is performed using the training subset corresponding to the key-frames SEI dataset. In this experiment, we aim to analyze and compare the results obtained from the generated models with the results of the models generated in the previous experiment. We note that while the key-frames SEI dataset has fewer frames than the normal dataset, its images should carry greater facial cues.
- **Experiment 3:** This experiment concerns the normal SEI dataset. Since it is unbalanced, with respect to the four classes, this experiment aims to determine whether or not the generated models, in Experiment 1, memorized the uneven class distribution of the corresponding training dataset. For this purpose, the corresponding test subset is supplied class by class, and not completely at once, to these models. The results are compared to those produced in Experiment 1.
- **Experiment 4:** This experiment concerns the balanced normal SEI dataset. It aims to understand how the different models would perform in a scenario where the subsets used are balanced with respect to the four classes. The best-performing models, from each architecture used in Experiment 1, are trained to generate corresponding models. These models are tested on the test subset of the balanced normal SEI test subset. The results are compared with those obtained in Experiment 1.

### 6.2. Experimental Parameters

In this study, the training protocol we used for all the models does not impose an upper limit on the number of epochs. Instead, a minimum threshold of 25 epochs is established to ensure adequate initial training. To optimize the training process and prevent overfitting, we employ an early stopping mechanism [43]. This mechanism monitors the validation performance, at each epoch, and suspends any further training if there is no improvement in the validation loss for the last 20 consecutive epochs. The model parameters corresponding to the epoch with the best validation performance (within the last 20 epochs) are retained. The early stopping approach ensures that the model is sufficiently trained, without using unnecessary computational burden. To summarize, the minimum epoch limit guarantees that the model has ample opportunity to learn from the data, while the early stopping mechanism helps in identifying, early on, the point of convergence, thereby avoiding overfitting.

To ensure consistency, all the deep learning hyper-parameters for training are kept consistent throughout all the experiments executed with the models used. These hyper-parameters are listed in Table 3, given below.

We note that all our experimental work, consisting of training and testing, is performed using NVIDIA A100 cloud GPUs.

### 6.3. Results and Discussion

In this subsection, we report on the results obtained from performing our comprehensive computational study. We also discuss the performance of the various architectures and models we used. The obtained results are summarized in Table 4, and from a quick glance at it, we can easily perceive that the methodology developed in this paper outperforms existing system surveyed the the literature.

In fact, we have conducted four types of computational experiments, and each experiment involves all or most of the models but with a different parts of the datasets discussed in the previous sections. The following three subsections comment on the results presented in Table 4 for each corresponding experiment. The last subsection gives global cross-comparison comments, insights, and some lessons learned.

It is worth noting at this point that globally our results showed that there are three models that performed best in our experiments, namely, the five-layer CNN, ResNet-18, and ViT-Small. So, in what follows, we present detailed results with these models for the relevant experiments.

#### 6.3.1. Experiment 1 Results

In Experiment 1, all models performed significantly better than most existing deep learning and machine learning models/systems used for tasks similar to the task of our paper. In particular, the ViTs and ResNets achieved diagnostic and classification accuracy scores that were close to perfect, attaining state-of-the-art scores. This observation holds true for most of the experiments conducted in our comprehensive study. On the other hand, the CNN models exhibited slightly lower accuracy performance. One hypothesis to explain these findings is the inherent architectural differences between the models. ViTs and ResNets incorporate advanced features such as self-attention mechanisms and skip connections, which might allow for more effective learning of global and less obvious features and patterns present in our SEI datasets. Furthermore, even though shallower than the models utilized conventionally, the ViT and ResNet architectures were significantly shallower than the CNN architectures and still had more learnable parameters.

It is interesting to note that an increase in a model’s size does not correlate with an increase in performance, as might intuitively be expected. For instance, in the case of the ResNets, the performance remains similar between ResNet-18 and ResNet-34, even though ResNet-34 has almost twice as many learnable parameters. Similarly, while there is a slight increase in performance between ViT-Tiny and ViT-Small, the ViT-Base actually has a lower, yet still appreciable, accuracy score.

There are two possible reasons for the above observation: First, the scores of the shallower models are already high due to effective data processing and the distilled information content being communicated (SEI images carry exactly the gait and, economically, not more). Consequently, deeper models cannot significantly outperform the shallower models as their capacity to capture more features is not needed. Second, the deeper models might not be learning new information but rather memorizing labels. However, this is unlikely since the observed drops in performance were negligible.

**Figure 10:**
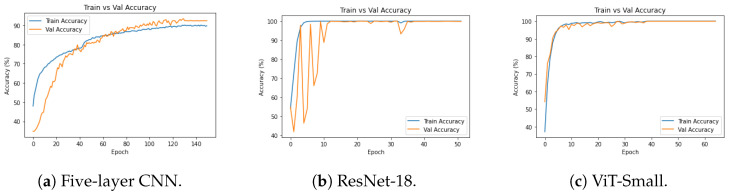
Experiment 1 training/validation accuracies for selected models.

**Figure 11:**
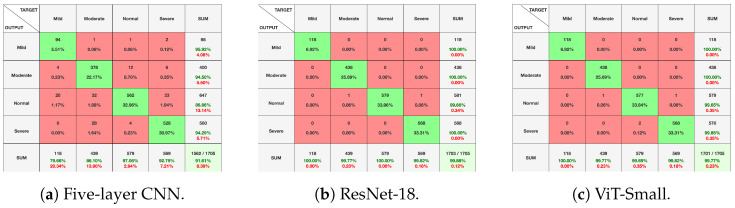
Experiment 1 confusion matrices for selected models.

**Figure 12:**
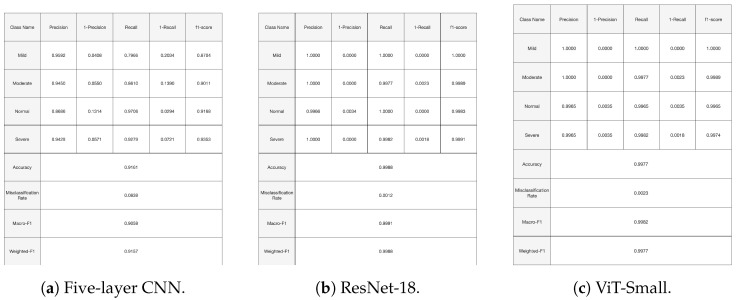
Experiment 1 classification reports for selected models.

Figure 10, Figure 11 and Figure 12, given above, respectively show for the models five-layer CNN, Resnet-18 and ViT-Small, the training and validation curves, the confusion matrices, and the classification reports. For these three models, the learning curves show interesting training and validation accuracies, at the end of the training period. Furthermore, the models are expected to have high generalization ability, as no overfitting is observed. The confusion matrices and classification reports confirm the good performance of the models when applied to the test dataset. Nevertheless, the five-layer CNN has low accuracy in detecting the mild class. In terms of the confusion matrices, Figure 11, the best performing models are the ResNet-18 and ViT-Small as they show near-perfect per-class percentages. The CNN five-layer model suffers a bit in that area, especially for the least represented mild class. These same observations are confirmed by the precision and recall figures given in the classification reports Figure 12.

#### 6.3.2. Experiment 2 Results

There is a considerable drop in the accuracy scores across the board in this second experiment. This decline can be attributed to the paucity of data when utilizing the key-frames SEI dataset. In fact, there were fewer frames available for extraction and training than in the case of the normal SEI dataset. This results in models unable to generalize effectively across the dataset. Unlike emotion detection, where a single key frame might provide sufficient information, this study necessitates multiple sequences of frames, even containing repeated ones, underscoring and highlighting the type of gait for the corresponding subject.

Once again, ResNets and ViTs outperformed the CNN architectures. However, several interesting patterns emerged that warrant further exploration. Firstly, while the performances of ViT-Tiny and ViT-Small are similar, ViT-Base performs significantly better than the two smaller models. One explanation for this observation is that ViT-Base, with its greater capacity and more complex architecture, might be better equipped to capture and utilize the temporal dependencies present in the time series data, leading to superior performance.

Secondly, we observe that the five-layer CNN performs much better than the six-layer CNN, which performs worse than random guessing. A possible explanation for this could be that the six-layer CNN is overfitting to the limited data available, leading to poor generalization. Alternatively, the additional layer might be disrupting the model’s ability to learn effective representations, suggesting that the optimal architecture for this task might be shallower.

Finally, both ResNet models achieved the highest levels of performance, with accuracy scores that are within a few percentage points of each other. However, the shallower ResNet-18 performs slightly better.

#### 6.3.3. Experiment 3 Results

In the third experiment, the patterns observed in the first experiment persisted, as expected. Notably, the ResNets and ViTs maintained the same weighted accuracies across both experiments. This consistency is a positive indication that these models did not memorize the labels of the images. In contrast, both CNN models experienced a slight drop in performance in the third experiment compared to the first. This decline could be attributed to the models, with their reduced number of parameters, attempting to memorize the class labels. However, overfitting did not appear to be a significant issue as the decrease in accuracy score was minor.

#### 6.3.4. Experiment 4 Results

In the fourth experiment, only the best-performing models from the previous three experiments were tested. As expected, all the learned models exhibited near-perfect accuracy, with ResNet-18 achieving a perfect accuracy. However, it is anticipated that ResNet-18 may exhibit some inaccuracies, as the size of the test data increases.

**Figure 13:**
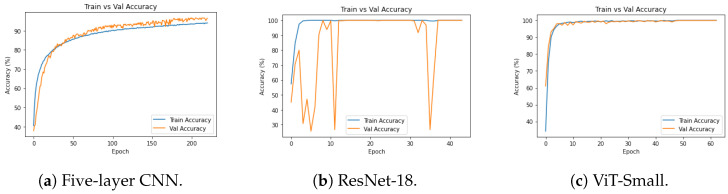
Experiment 4 training/validation accuracies for selected models.

**Figure 14:**
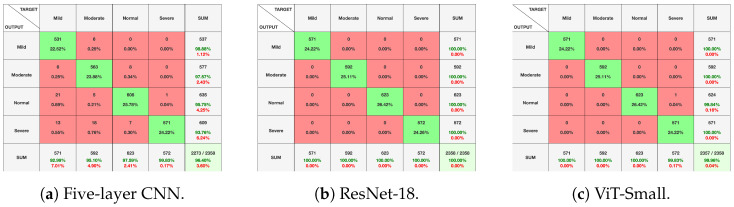
Experiment 4 confusion matrices for selected models.

**Figure 15:**
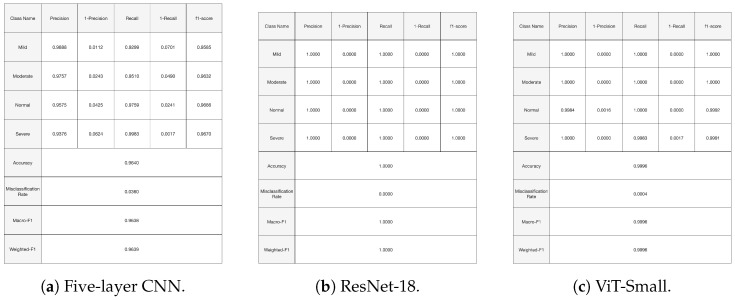
Experiment 4 classification reports for selected models.

Figure 13 shows the training and validation curves for the top three performing models. At the end of the training period, all models present significant training and validation accuracies. Moreover, Figure 14 and Figure 15 present the confusion matrices and the classification report for the three models. All the evaluation criteria values, precision, recall, and F1-score, are significant for all classes for the Vit-Small and ResNet-18 models. For the five-layer CNN, the evaluation criteria values are lower than for the other models. The accuracy for the least represented mild class improved a bit with the oversampling we used in this experiment.

In particular, the performance of the three models is interesting compared the state of the art. Indeed, in [7], a machine learning technique using a gait-based feature engineering approach was applied to the dataset we used in our paper. A maximum accuracy of 97% was obtained with a Random Forest classifier with 250 trees. Moreover, in [26], an overall classification accuracy of 71.25% was achieved using a complex approach that is entirely based on deep learning applied to a locally collected controlled dataset comprising 54 Parkinson’s Disease patients and 26 healthy subjects.

#### 6.3.5. Overview of Results of Experiments

Across all experiments, ResNet-18 consistently demonstrates superior accuracy performance. ResNet-18, although less complex and less computationally intensive, outperforms Vision Transformers (ViTs), which demand even greater computational resources. Since the system is envisioned for use in a medical setting, where accuracy is of paramount importance, the ResNet architecture would be best suited for this application.

The residuals and the bypass connections, which are the backbone of the ResNet architecture and seem to capture even the faint patterns, could be essential in the accuracy scores attained by the architecture. In comparison, Vision Transformers (ViTs) require extensive computational resources due to their self-attention mechanisms, which capture long-range dependencies in the data. While ViTs excel in capturing global context, they often require larger datasets and more computational power to train effectively. Due to the structure of energy images, many patches input to the ViT were completely or almost completely white, with limited features to extract. Thus, the model would likely have performed better with a larger dataset.

As a summary and a global overview of the results of the four experiments we conducted, it is worth mentioning that, while the choices of models used are definitely important in delivering the results displayed, the approach has proven good robustness and promises to be the basis for an applicable PD classification framework. Firstly, our approach, does not rely on any specific feature engineering method or on an any precise definition of gait; it rather generates SEIs from sequences of almost any contiguous 17 frames from the raw videos. In many ways, this SEI synthetization technique functions as an effective dataset enhancement method that is somehow expanding the size of the dataset and enhancing the generalization capabilities and robustness of the models. This can be easily seen by overviewing the results of experiments 1, 2, 3, and 4 in the following manner.

Recall that Experiment 1 used the normal SEI dataset, while Experiment 2 used the more restricted key-frame SEI dataset, based on generating SEIs that start with a key frame. The key-frame SEI dataset has far many fewer SEI frames than the Experiment 1 dataset. However, both are based on the same original video dataset. The results of Experiment 2 are inferior to those of the near-perfect Experiment 1 results. We have further attempted to confirm the results of Experiment 1 by performing Experiment 3 on the same normal SEI dataset, and supplying the classes one by one to the model in the testing phase. This allowed us to confirm that the results of Experiment 1 did not hinge on any model perception of the class distributions. This definitely proved that our SEI sythetization approach is working, and that the greater number of SEIs generated with our approach, not originating just from key frames, enhanced the generalization capabilities of our models. This has been further confirmed by Experiment 4, which performed more data augmentation using a standard random oversampling technique to achieve some class balancing. In fact, Experiment 4 enhanced class accuracies, especially for the under-represented classes.

In retrospect, the SEIs generated based on skeletons present a powerful motion abstraction technique, giving the model a way of perceiving the motion of the person’s body independent of the original video. Our method for generating the SEIs, by not limiting them to start from a key frame, captures even more motion patters and enhances the generalization capabilities, as proven with the test datasets results for the four experiments.

## 7. Conclusions and Perspectives

In this study, we successfully devised a deep learning-based approach to detect and classify the severity of Parkinson’s Disease utilizing an open-source, relatively large dataset of videos recorded in diverse and uncontrolled environments and labeled according to the well-accepted UPDRS standard. We applied an automatic workflow based on state-of-the-art pose estimation techniques to the dataset of videos to generate Skeleton Energy Images to effectively capture the gaits of the subjects. Then, we devised a comprehensive and systematic set of experiments to train and test our carefully selected set of advanced deep learning models. The results demonstrated highly accurate classification of the videos of patients, achieving state-of-the-art performance.

Furthermore, the good results shown by the models presented in this paper can be used as a basis for the development of software that can made available in the offices of doctors treating PD patients. By placing a few regular cameras strategically in a given doctor’s office, the software can capture a patient as he/she walks in and can be used to alert and advise the doctor as to how the patient is responding to a given treatment. We see this as practical, since our approach for synthesizing SEIs can be easily applied to any subset of videos. In fact, recent transfer learning techniques/experiences [44] in similar fields and with similar models could be leveraged to perform model fine-tunings to specific perimeters and conditions.

In fact, we believe the above ideas carry good prospects for improving the lives of many patients, especially in areas with limited financial capabilities. However, a regulated study needs to be performed to prove the generalization capabilities of our selected models and to seek further enhancement directions for our methodology’s workflows. Moreover, creating a large video dataset of patients with Parkinson’s Disease is important to the field and could prompt a revisit to this study. Furthermore, our proposed methodology, which is based on gait analysis, can be extended to detect many other neurological diseases such as Alzheimer’s disease, Huntington’s disease, etc. Thus, applying it to other neurological diseases is one of our future perspectives.

In future studies, we aim to realize the above and to generalize our framework to a broader range of video-based detection tasks that necessitate continuous data, such as sports analysis.

## Figures and Tables

**Table 1:** Selected architectures/models.

Architecture	Model Name	Model Parameter
**CNN**	CNN-5	5 Conv. Layers
	CNN-6	6 Conv. Layers
**ResNet**	ResNet-18	Depth 18
	ResNet-34	Depth 34
**ViT**	Vit-Tiny	Tiny Patch
	Vit-Small	Small Patch
	Vit-Base	Base Patch

**Table 2:** Number of SEIs in datasets/subsets employed in experiments.

	Total	Training	Validation	Testing
**Normal SEI Dataset**	17,038	13,628	1705	1705
**Key-Frame SEI Dataset**	513	410	51	52
**Balanced Normal SEI Dataset**	23,580	18,864	2358	2358

**Table 3:** Hyper-parameters for deep learning models used for experiments.

Hyper-Parameter	Value
Weight Initialization	Random
Optimizer	AdamW
Loss Function	Cross Entropy Loss
Learning Rate	5 × 10^−5^
Batch Size	64

**Table 4:** Accuracy scores for corresponding generated model instances.

Models	Experiment 1	Experiment 2	Experiment 3		Experiment 4
			**Average**	**Weighted Average**	
**ViT-Tiny**	99.71%	32.69%	99.74%	99.71%	
**ViT-Small**	99.76%	32.69%	99.81%	99.76%	99.96%
**ViT-Base**	99.70%	59.63%	99.43%	99.70%	
**ResNet-18**	**99.88%**	**67.30%**	**99.89%**	**99.88%**	**100.00%**
**ResNet-34**	**99.88%**	63.46%	**99.89%**	**99.88%**	
**CNN 5-layer**	91.61%	30.77%	88.41%	90.73%	96.40%
**CNN 6-layer**	87.22%	7.69%	67.86%	82.99%	

## Data Availability

The raw data supporting the conclusions of this article will be made available by the authors on request.
